# Points

**Published:** 1887-02

**Authors:** 


Staph.—Escape of hot flatus, smelling like rotten eggs. (E. A. F.)

Kali phos.—Similar mental symptom to Kali brom.

Zincum met.—Stitch at the upper part of sternum, extending into left lumbar region, with dread of stooping. (R. R. Gregg.)

